# A Randomised, Double-Blinded, Placebo-Controlled, Parallel Study of Vitamin D3 Supplementation with Different Schemes Based on Multiples of 25,000 IU Doses

**DOI:** 10.1155/2013/327265

**Published:** 2013-01-28

**Authors:** Etienne Cavalier, Werner Faché, Jean-Claude Souberbielle

**Affiliations:** ^1^Department of Clinical Chemistry, University of Liège, CHU Sart-Tilman, 4000 Liège, Belgium; ^2^Zuidstationstraat, Gent, Belgium; ^3^Laboratoire d'Explorations Fonctionnelles, Hôpital Necker-Enfants Malades, 75015 Paris, France

## Abstract

Vitamin D (VTD) treatment is recommended in patients presenting different causes of diseases. To treat these patients, physicians rely on the different available pharmaceutical forms present in their country. Unfortunately, even in a given country, there is no consensus on the best way to treat the patients. In Belgium, VTD is mostly prescribed as ampoules containing 25,000 IU of VTD. In this randomised controlled study, we evaluated whether four therapeutic schemes using multiples of 25,000 IU of VTD according to basal vitamin D concentration were able to increase or maintain the 25(OH)D serum level above 30 ng/mL. We randomized 175 subjects who received the drug (*n* = 140) or placebo (*n* = 35). Total duration of the study was 12 weeks. Doses ranged from 4167 to 1667 IU/day. Blood sampling was performed at baseline and each 4 visits. In the treated (placebo) subjects, mean 25(OH)D serum concentration was 18.7 (19.1) ng/mL at baseline and 31.5 (20.7) ng/mL at w-12. At the end of the study, 57.1% of the subjects treated with VTD presented 25(OH)D serum concentration ≥30 ng/mL, whereas 94.3% were ≥20 ng/mL. In conclusion, the doses administered were safe and increased or maintained the 25(OH)D concentration ≥20 ng/mL. However, concentrations ≥30 ng/mL were only achieved in 57.1% of the subjects.

## 1. Introduction

Vitamin D deficiency has recently been identified as a worldwide problem [1]. Indeed, the dietary sources of vitamin D are scarce, the only really significant ones being marine fatty fish, although egg yolk and some mushrooms could be additional sources [2]. Thus, in humans, the major source of vitamin D comes from the exposure of the skin to sunlight. While, in tropical zones, UVB radiation is sufficient throughout the year, it is absent for a significant part of the year in more northern (or southern) latitudes. In Belgium (around 51° North), the UVB ray will be insufficient to allow the skin synthesis of vitamin D during approximately 6 months of the year. Hence, while all experts agree that the 25-hydroxy vitamin D (25(OH)D) level is the correct indicator of vitamin D status, they do not agree on the 25(OH)D level that should be used to define vitamin D sufficiency. Indeed, the expert panels from the Endocrine Society [3] or the Institute of Medicine (IOM) [4] have recently released divergent recommendations on the use of vitamin D. The recent report by the IOM indicates that a 25(OH)D level of 20 ng/mL is largely sufficient and the RDIs set forth should be sufficient for 97.5% of the population to achieve that level whereas the Endocrine Society group considers that the optimal 25(OH)D level for musculoskeletal health should be 30 ng/mL and more in individual patients. It should be considered that the IOM cutoff is intended for public health recommendations while the Endocrine Society group targets its recommendations on patient care and considers that vitamin D deficiency (what should be avoided in any patient) corresponds to 25(OH)D levels <20 ng/mL and insufficiency (what may be deleterious for a significant proportion of patients) to levels of 20 to 30 ng/mL. Now, considering a threshold of 30 ng/mL, but even with the more conservative IOM cutoff of 20 ng/mL, insufficient vitamin D status is highly frequent. This can easily be understood if one considers that the usual daily intakes of vitamin D are not higher than 200 IU (and often much lower) [5, 6] whereas the most conservative recommendations of the IOM are of 600 IU for adults up to 70 years and 800 IU for those older. Taking that into consideration, one might consider that a moderate supplementation without prior evaluation of vitamin D status is necessary at the population level to fulfil this recommendation. Now, there are some groups of patients for whom many experts consider that a certain reasonably evidence-based range of 25(OH)D concentration should be targeted for optimal effects of vitamin D. In these patients, we [7], like many others [3, 8] consider that 25(OH)D should be measured, and vitamin D supplementation should be prescribed according to the measured serum level (higher dosages in those with lower 25(OH)D concentrations). This mostly apply to musculoskeletal effects of vitamin D and concern patients with, or at risk of, osteoporosis, chronic kidney disease, primary hyperparathyroidism, intestinal malabsorption, and so on… for whom we use to target concentrations of 30–60 ng/mL. Daily dosages (600 to 4000 IU/day for example) may be preferred as they are theoretically more physiologic, but they usually suffer from poor compliance. Although it has become usual practice to avoid very large spaced out doses such as 500,000 IU vitamin D3 once a year due to the results of a recent RCT [9], many physicians and patients prefer intermediate doses given at intervals between one week and one to two months. In Belgium, one of the major source of pharmaceutical vitamin D supplementation is the D-Cure (SMB Laboratories, Belgium), an oily solution containing 25,000 IU of cholecalciferol per ampoule. This drug is largely prescribed to correct vitamin D deficiency, but there is actually no validated treatment scheme. Several protocols using doses of 50,000 IU vitamin D2 or D3 [1, 8] or 100,000 IU vitamin D3 [10] have been tested, but to our knowledge, only one paper has reported the results of supplementation with doses of 25,000 IU vitamin D3 [11]. In this randomised, parallel, double-blinded, placebo-controlled study, we aimed to evaluate whether four therapeutic schemes that used different doses of D-Cure according to basal vitamin D concentration were able to increase or maintain (depending on the baseline level) the 25(OH)D serum level above 30 ng/mL.

## 2. Methods

### 2.1. Methodology

The study took place between the 23rd of January and the 22nd of July, 2011. More than two hundred subjects were screened within 14 days prior to starting the study. Those who met all inclusion and none of the exclusion criteria were randomized to one of the four strata based on their baseline 25(OH)D serum concentration. The subjects took the study medication under the supervision of the study personnel at Visit 2 (week 0), Visit 3 (week 2), Visit 4 (week 4), and Visit 5 (week 8). The total duration of the study was 12 weeks with an 8-week period of supplementation followed by a 4-week period without supplementation. Blood samples for measurement of 25(OH)D concentration, calcium, phosphorus, and albumin were collected at each visit after an overnight fast and before next the vitamin D dose. We included Caucasian (defined as European and North African) male or female subjects over 50 years old with a body mass index between 18 kg/m^2^ and 35 kg/m^2^ who were able to comply with all study procedures. They gave written, informed consent to participate in this trial. The exclusion criteria were past or current history of any immunological, neoplasic, endocrine, haematological, hepatic, renal, gastrointestinal, neurological, or psychiatric abnormalities or medical disease. Subjects who used UV light solarium 2 weeks before the screening visit or planned to travel outside European countries during the study were excluded. We also excluded patients under treatment with drugs that may interfere with vitamin D metabolism (e.g., phenobarbital, phenytoin, and glucocorticoids) and those with past or current history of granulomatosis, especially sarcoidosis, urinary lithiasis, and osteomalacia. Finally, patients who presented a 25(OH)D concentration >60 ng/mL, serum creatinine >150 *μ*mol/L and albumin corrected serum calcium >2.65 mmol/L (corresponding to 10.6 mg/dL) at screening were also excluded, as well as those with any sensitivity or allergy to any of the products used in the study or a history of drug and/or alcohol abuse.

#### 2.1.1. Randomisation

At the baseline/randomisation visit (Visit 2), after confirmation that the patients met all eligibility criteria for the study, they were randomized and assigned a randomisation number by an interactive web response System (IWRS). A central, electronic, balanced, permuted blocks randomisation were used. Patients were assigned at random to placebo or vitamin D treatments of the 4 possible groups. The IWRS will randomize a subject to either test or placebo according to a 4 : 1 ratio. It was a block randomization (blocks size of 5) stratified by subgroup.

The objective of this randomization was to obtain 50 patients in each group (40 to D-CURE treatment and 10 to placebo). When the objective was attained in a group, no more inclusion was accepted in that group. Finally, only the objective in group with 25(OH)D level >30 ng/mL was not attained. In fact, only 20 patients were randomized to receive vitamin D and 5 to placebo in that group, due to the fact that the patients with 25(OH) level >30 ng/mL are rare in the Belgium population. In total, 175 patients have been enrolled (140 in treatment group and 35 in the placebo group). The reasons of screening failures were BMI > 35 (exclusion criteria; 1 subject), patient consent withdrawn (1 subject), HCV positive (1 subject), and hypothyroidism (1 subject).

#### 2.1.2. Treatment Scheme

At the end of the screening phase, 175 subjects were selected. 140 were assigned to receive the D-Cure and 35, the placebo according to this scheme received.

Group 1. Subjects with baseline serum concentrations of 25(OH)D ≤ 10 ng/mL. Intake: 3 ampoules taken at week 0 and 2 followed by 2 ampoules at week 4 and 8 (total intake: 250,000 IU; *n* = 40) or placebo (*n* = 10).
Group 2. Subjects with baseline serum concentrations of 25(OH)D > 10 ng/mL and ≤20 ng/mL. Intake: 3 ampoules taken at week 0 followed by 2 ampoules at week 2 and 1 ampoule at week 4 and 8 (total intake: 175,000 IU; *n* = 40) or placebo (*n* = 10).
Group 3. Subjects with baseline serum concentrations of 25(OH)D > 20 ng/mL and ≤30 ng/mL. Intake: 2 ampoules taken at week 0 followed by 1 ampoule at week 2, 4, and 8 (total intake: 125,000 IU; *n* = 40) or placebo (*n* = 10).
Group 4. Subjects with baseline serum concentrations of 25(OH)D > 30 ng/mL and ≤60 ng/mL. Intake: 1 ampoule taken at week 0, 2, 4, and 8 (total intake: 100,000 IU; *n* = 20) or placebo (*n* = 5).

The treatment schemes are summarized in Table 1. These schemes were chosen on the basis of the relationship between a received daily dose of vitamin D3 and the subsequent increase in the 25(OH)D serum level that had been published previously. As, according to a rule of thumb, a daily dose of 1,000 IU vitamin D3 was considered to increase the 25(OH)D level by approximately 7 to 10 ng/mL [12], we decided to give cumulative doses over the 8-week period of supplementations (60 days) that were approximately equivalent to 4,000, 3,000, and 2,000 IU/day in those with a baseline 25(OH)D concentration <10 ng/mL, between 10 and 20 ng/mL, and between 20 and 30 ng/mL, respectively. As we were using 25,000 IU doses, this translated in practice into 250,000 IU over the 8-week period, corresponding to 4167 IU/day in group 1 subjects, 175,000 IU corresponding to 2917 IU/day in group 2, and 125,000 IU corresponding to 2083 IU/day in group 3. For group 4 subjects, we arbitrarily decided to give one ampoule every other week corresponding to 1667 IU/day. For practical reasons, the number of takings of vitamin D3 ampoules was limited to four, so that the study personnel was able to be present at each taking, ensuring an optimal compliance.

We used the DiaSorin Liaison (Stillwater, MN, USA) for 25(OH)D determination. In our lab, intra-, and interassay coefficient of variation are <5% and <10%, respectively. The functional detection limit is 8 ng/mL serum calcium; phosphorus and albumin concentrations were evaluated with the Roche Modular instrument (Mannheim, Germany).

### 2.2. Statistical Methods

We used the raw SAS Version 9.1 or higher software to compute the results. All statistical tests were performed two sided, and a *P* value less than 5% was considered as statistically significant. Mean changes from baseline to week 12 was calculated with ANOVA. The percentages of subjects reaching 25-hydroxyvitamin D serum concentrations ≥20 ng/mL and ≥30 ng/mL at the end of the study were analysed by means of a chi-square test comparing D-CURE and placebo. For the main continuous baseline characteristics age, and BMI, an ANOVA was performed, and for the binomial baseline variable sex, we used a Cochran-Mantel-Haenszel Test. All safety data obtained in this study was tabulated with descriptive statistics. Comparisons between treatment groups were based on descriptive statistics.

This trial was approved by the relevant Independent Ethics Committee (IEC) and the Competent Authority prior to the start of the study and conducted in compliance with the protocol, with the ICH Harmonized Tripartite Guideline, Guideline for Good Clinical Practice, Step 5 (CPMP/ICH/135/95), the applicable regulatory requirements based on EU Directive 2001/20/EC and EU GCP Directive (2005/28/EC), and the Declaration of Helsinki (World Medical Association) in its revised edition.

## 3. Results

One hundred and seventy-five subjects completed the study. Their demographics are shown in Table 2. As treatment was taken under the supervision of the study personnel, observance was of 100%.

In the treated subjects, the mean 25(OH)D serum concentration was 18.7 ng/mL at baseline and 31.5 ng/mL at week 12, with a mean change to baseline of 12.9 ± 10.7 ng/mL. In the placebo group, the mean 25(OH)D serum concentration was 19.1 ng/mL at baseline and 20.7 ng/mL at week 12; the mean change to baseline was 1.7 ± 6.3 ng/mL. The mean 25(OH)D serum concentration at all timepoints is shown in Table 3 for each group. There was a significant increase in 25(OH)D levels at week 12 for all treated groups compared to baseline (19.7 ± 9.4, 14.8 ± 8.5, 9.5 ± 8.9, and 2.45 ± 9.8 ng/mL for the Group 1, 2, 3, and, 4, resp.). During the study there was a significant difference between the vitamin D and placebo groups in the 25(OH)D concentrations at week 2, 4, 8, and 12 (*P* < 0.001 for the global group analysis).

The highest serum 25(OH)D concentration (68 ng/mL) was observed at week 12 in one subject of the group 4, whose basal level was of 48 ng/mL.

**Figure 1:**
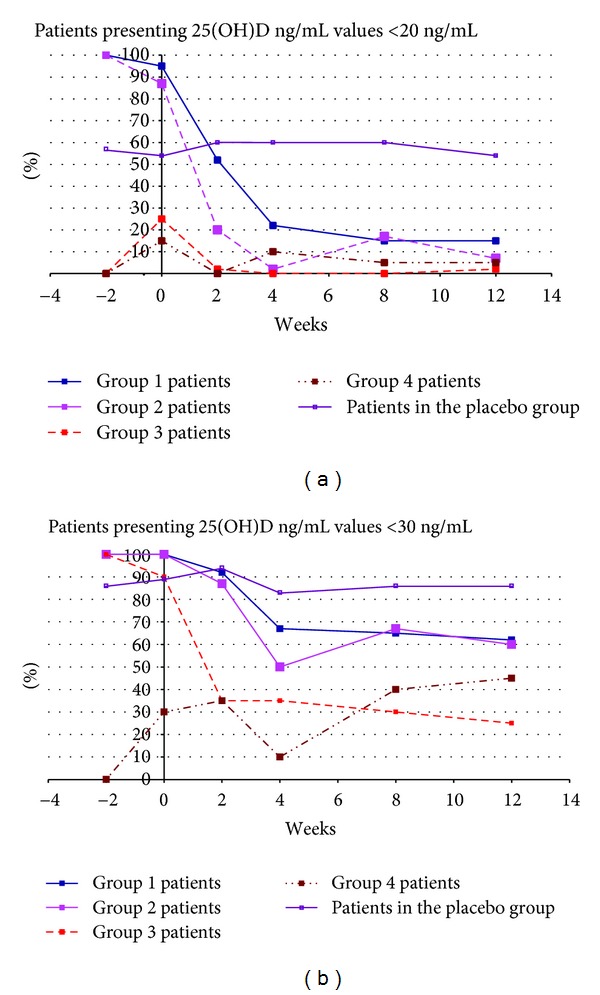
Evolution of the percentages of patients presenting 25(OH)D values <20 and <30 ng/mL throughout the study, according to their treatment scheme.

At the end of the study, (week 12) 57.1% (*n* = 80) of subjects in the vitamin D group had a 25(OH)D serum concentration ≥30 ng/mL, and 94.3% (*n* = 132), a concentration ≥20 ng/mL. In the placebo group, 20.0% (*n* = 7) had a 25(OH)D serum concentration ≥30 ng/mL and 54.3% (*n* = 19) ≥20 ng/mL. The number of subjects that did not reach 25(OH)D levels of 30 ng/mL or 20 ng/mL at each timepoint is shown in Table 3 and Figure 1 for each treated and placebo group separately. The lower number of subjects that did not reach 25(OH)D serum concentrations of ≥30 ng/mL at week 12 was seen in group 3 subjects (25%, *n* = 10/40). There were 25/40 (62%) subjects and 24/40 (60%) subjects who did not reach serum concentrations of 25(OH) vitamin D ≥30 ng/mL at week 12 in the groups 1 and 2, respectively.

Altogether 106 of the 140 subjects of the vitamin D group (75.71%) and 11 of the 35 subjects treated with placebo (31.43%) had a serum 25(OH)D concentration of ≥30 ng/mL at one or more time points during the study. The highest number of subjects was in subgroup 3. In this group, 97.5% of the subjects achieved a serum 25(OH)D concentration of ≥30 ng/mL in one or more visit, but only 77.5% of them had a serum 25(OH)D concentration of ≥30 ng/mL at the end of the study.

There were no significant changes to baseline seen in the mean plasma phosphorus and calcium concentrations during the study. The mean change to baseline at week 12 for plasma phosphorus was −0.29 mg/dL (±1.960) in subjects treated with SMB D-CURE and −0.26 mg/dL (±0.584) in the placebo group. The mean change to baseline at week 12 for plasma calcium was 0.00 mg/dL ± 0.378 in subjects treated with SMB D-CURE and 0.02 mg/dL ± 0.259 in the placebo group. We did neither observe hypercalcemia nor kidney stone during the study.

## 4. Discussion

We performed a trial to test several schemes of supplementation with ampoules containing 25,000 IU vitamin D3 in subjects 50 years of age and older, based on their baseline serum concentration of 25(OH)D. The main strengths of this study are its double-blinded, placebo-controlled nature, allowing to control for possible confounders such as season-, or diet-related changes in the 25(OH)D level, and the supervision by the study staff of the administration of all vitamin D3 doses allowing for a compliance of 100%.

The concentration of 25(OH)D in serum was significantly increased after treatment with D-CURE compared to placebo for the total analysis. As this study took place during a part of spring, it is important to note that we did not observe any significant increase in the placebo group, suggesting that these increases in the treated group are only due to the treatment.

At week 12 of the study, that is one month after a 60-day supplementation period with doses of vitamin D3 corresponding to 1667 to 4167 IU/day, the mean change from baseline in the different subgroups ranged from +2.5 to +19.7 ng/mL. This was somewhat lower than the expected increase of 7 to 10 ng/mL in the 25(OH)D level for every 1,000 IU vitamin D3 per day, according to Heaney [12]. The 25(OH)D increase in our study was also lower than what could have been predicted from a more recent meta-analysis where the average increase during different RCT or open trials was 0.78 ng/mL per 40 IU of vitamin D3 supplement per day in Caucasian subjects over 50 yr old, albeit with a great variability [13]. In this meta-analyse, the authors underlined different sources of between-trial variability. Poor adherence to supplementation, often due to coadministration of calcium supplements, use of vitamin D2 instead of vitamin D3, and high proportion of overweight subjects included in the trial were identified as potential causes for a weak increase in the 25(OH)D serum level. These reasons do not apply to the present study in which compliance was 100%, calcium supplements were not provided, vitamin D3 was used, and the mean BMI was close to 26 kg/m^2^ with very few obese (BMI > 30) subjects. Another potential cause of variability in the 25(OH)D response to vitamin D supplementation among studies is the use of various 25(OH)D assays. In the present study, we used the DiaSorin Liaison assay, an automated assay widely used worldwide and for which the results of the external proficiency testing DEQAS samples in our laboratory are very close of the all laboratory trimmed mean (ALTM).

While most of the vitamin D-treated subjects reached a 25(OH)D serum concentration of 20 ng/mL or more, only 42.5% of those with baseline concentrations below 10 ng/mL, 47.5% of those with baseline concentrations of 10 to ≤20 ng/mL, and 77.5% of those with a baseline level of 20 to ≤30 ng/mL achieved a 25(OH)D concentration of ≥30 ng/mL at week 12. Furthermore, only 65.0% of those who had concentrations of ≥30 ng/mL at week 0 had still such a value at week 12 after treatment. Overall, this suggests that the doses of vitamin D3 administered in the present study were insufficient to achieve or maintain the 30 ng/mL target in a significant proportion of the included subjects. It must be underlined that the dose administered to the subjects with a baseline serum level ≤10 ng/mL was already very close (slightly above in fact) to the upper safety limit (UL) of 4,000 IU/day defined by the IOM [4]. Thus, if a 25(OH)D level of 30 ng/mL or more is targeted in future studies, higher doses than the IOM UL should be used.

At week 12, change from baseline in the 25(OH)D concentration of the placebo groups ranged from −8.20 to +4.30 ng/mL. The negative change was observed in the subgroup with baseline 25(OH)D > 30 ng/mL and confirms that even if this target level is reached, a maintenance dose is usually required to sustain the serum 25(OH)D above 30 ng/mL.

The maximum serum 25(OH)-vitamin D concentration (68 ng/mL) was observed at week 12 and is far from 150 ng/mL, generally considered as the potentially “toxic” limit. We did not observe clinically significant change in the plasma calcium and phosphorus concentrations.

In conclusion, this study explored the change in serum 25(OH)D concentrations in subjects with different baseline concentrations of 25(OH)D who received different doses of vitamin D. The current doses administered were safe and produced a significant change compared to placebo, increasing or maintaining (depending on the baseline level) the 25(OH)D concentration ≥20 ng/mL. However, serum concentrations of 25(OH)D ≥ 30 ng/mL were only achieved in 57.1% of the subjects receiving the vitamin D, indicating that the doses may need to be increased in subsequent studies where this concentration will be targeted.

## Figures and Tables

**Table 1:** Treatment scheme with different intakes of vitamin D according to the 25(OH)D basal level of the subjects.

	Week 0	Week 2	Week 4	Week 8	Total
Group 1					
≤10 ng/mL					
Treated group (*n* = 40)	75,000 UI	75,000 UI	50,000 UI	50,000 UI	250,000 UI
Placebo (*n* = 10)	placebo	placebo	placebo	placebo	placebo
Group 2					
>10 ng/mL and ≤20 ng/mL					
Treated group (*n* = 40)	75,000 UI	50,000 UI	25,000 UI	25,000 UI	175,000 UI
Placebo (*n* = 10)	placebo	placebo	placebo	placebo	placebo
Group 3					
>20 ng/mL and ≤30 ng/mL					
Treated group (*n* = 40)	50,000 UI	25,000 UI	25,000 UI	25,000 UI	125,000 UI
Placebo (*n* = 10)	placebo	placebo	placebo	placebo	placebo
Group 4					
>30 ng/mL and ≤60 ng/mL					
Treated group (*n* = 20)	25,000 UI	25,000 UI	25,000 UI	25,000 UI	100,000 UI
Placebo (*n* = 5)	placebo	placebo	placebo	placebo	placebo

**Table 2:** Demographic data of the 175 subjects (140 treated with different vitamin D doses according to the basal level and 35 with placebo) included in the study. For the main continuous baseline characteristics age, and BMI, an ANOVA was performed, and for the binomial baseline variable sex, we used a Cochran-Mantel-Haenszel test.

	Vitamin D treatment group					Placebo group					*P* value
	Group 1 (*n* = 40)	Group 2 (*n* = 40)	Group 3 (*n* = 40)	Group 4 (*n* = 20)	Subtotal (*n* = 140)	Group 1 (*n* = 10)	Group 2 (*n* = 10)	Group 3 (*n* = 10)	Group 4 (*n* = 5)	Subtotal (*n* = 35)
**Age (years)**											

Mean ± SD	65.8 ± 10.9	62.5 ± 9.7	64.6 ± 11.1	64.8 ± 10.5	64.4 ± 10.6	69.7 ± 14.4	65.0 ± 11.7	58.4 ± 6.0	65.2 ± 13.4	64.5 ± 11.9	0.757
Min–max	51–89	50–84	51–88	52–87	50–89	50–90	50–85	51–71	50–81	50–90	

**Subject gender**											

Male: *n* (%)	14 (35.0)	15 (37.5)	17 (42.5)	5 (25.0)	51 (36.4)	5 (50.0)	5 (50.0)	4 (40.0)	1 (20.0)	15 (42.9)	0.473
Female: *n* (%)	26 (65.0)	25 (62.5)	23 (57.5)	15 (75.0)	89 (63.6)	5 (50.0)	5 (50.0)	6 (60.0)	4 (80.0)	20 (57.1)	

**BMI (kg/m²)**											

Mean ± SD	26.5 ± 4.6	26.2 ± 3.3	25.6 ± 3.5	25.8 ± 4.9	26.0 ± 4.0	27.7 ± 3.3	28.2 ± 5.4	24.8 ± 3.1	25.9 ± 2.8	26.7 ± 4.0	0.735
Min–max	19–35	20–34	19–34	19–37	19–37	22–32	20–35	19–30	24–31	19–35	

**Table 3:** 25(OH)D serum concentration and number (%) of subjects who did not reach the target levels of 20 ng/mL and 30 ng/mL at all time points.

		Visit 1 (week 2)	Visit 2 (week 0)	Visit 3 (week 2)	Visit 4 (week 4)	Visit 5 (week 8)	Visit 6 (week 12)
Group 1	25(OH)D Mean ± SD (min–max)	7.62 ± 2.11 (4.0–12.0)	9.08 ± 3.37 (4.0–19.0)	21.00 ± 5.97 (11.0–46.0)	26.93 ± 7.54 (14.0–45.0)	28.25 ± 8.31 (10.0–45.0)	28.63 ± 8.08 (13.0–49.0)
	*n* (%) with 25(OH)D ≤ 20 ng/mL	40 (100)	38 (95)	21 (52)	9 (22)	6 (15)	6 (15)
	*n* (%) with 25(OH)D ≤ 30 ng/mL	40 (100)	40 (100)	37 (92)	27 (67)	26 (65)	25 (62)
Group 2	25(OH)D Mean ± SD (min–max)	13.72 ± 2.63 (8.0–20.0)	15.30 ± 3.38 (7.0–23.0)	25.55 ± 5.89 (12.0–41.0)	31.65 ± 7.60 (18.0–50.0)	28.15 ± 7.69 (14.0–46.0)	30.10 ± 8.15 (13.0–50.0)
	*n* (%) with 25(OH)D ≤ 20 ng/mL	40 (100)	35 (87)	8 (20)	1 (2)	7 (17)	3 (7)
	*n* (%) with 25(OH)D ≤ 30 ng/mL	40 (100)	40 (100)	35 (87)	20 (50)	27 (67)	24 (60)
Group 3	25(OH)D Mean ± SD (min–max)	24.88 ± 2.75 (21.0–30.0)	24.38 ± 5.31 (12.0–38.0)	32.88 ± 5.63 (20.0–51.0)	33.90 ± 8.24 (24.0–62.0)	35.25 ± 7.22 (23.0–54.0)	33.90 ± 8.21 (5.0–50.0)
	*n* (%) with 25(OH)D ≤ 20 ng/mL	0 (0)	10 (25)	1 (2)	0 (0)	0 (0)	1 (2)
	*n* (%) with 25(OH)D ≤ 30 ng/mL	40 (100)	36 (90)	14 (35)	14 (35)	12 (30)	10 (25)
Group 4	25(OH)D Mean ± SD (min–max)	38.21 ± 6.11 (31.0–51.0)	32.70 ± 10.01 (11.0–53.0)	34.85 ± 8.25 (21.0–56.0)	35.65 ± 10.49 (17.0–55.0)	36.25 ± 11.90 (20.0–67.0)	35.15 ± 13.37 (11.0–68.0)
	*n* (%) with 25(OH)D ≤ 20 ng/mL	0 (0)	3 (15)	0 (0)	2 (10)	1 (5)	1 (5)
	*n* (%) with 25(OH)D ≤ 30 ng/mL	0 (0)	6 (30)	7 (35)	2 (10)	8 (40)	9 (45)
Placebo group (group 5 + 6 + 7 + 8)	25(OH)D Mean ± SD (min–max)	18.83 ± 10.89 (6.0–50.0)	19.09 ± 9.43 (5.0–42.0)	18.57 ± 8.48 (6.0–43.0)	19.06 ± 9.34 (6.0–39.0)	19.17 ± 9.41 (4.0–38.0)	20.74 ± 9.48 (4.0–39.0)
	*n* (%) with 25(OH)D ≤ 20 ng/mL	20 (57)	19 (54)	21 (60)	21 (60)	21 (60)	19 (54)
	*n* (%) with 25(OH)D ≤ 30 ng/mL	30 (86)	31 (89)	33 (94)	29 (83)	30 (86)	30 (86)
